# 2,9-Bis(1,3-benzothia­zol-2-yl)-1,10-phenanthroline dichloro­methane disolvate

**DOI:** 10.1107/S1600536808019995

**Published:** 2008-08-30

**Authors:** Jesmin Akther, Sergey Lindeman, Mohammad Rezaul Karim

**Affiliations:** a Tennessee State University, Department of Chemistry, 3500 John A Merritt Boulevard, Nashville, TN 37209, USA; b Department of Chemistry, Marquette University, PO Box 1881, Milwaukee, WI 53201-1881, USA

## Abstract

In the title compound, C_26_H_14_N_4_S_2_·2CH_2_Cl_2_, the two pendant benzothia­zole groups are slightly twisted with respect to the phenanthroline core [dihedral angles = 1.03 (7) and 9.05 (5)°]. Weak inter­molecular C—H⋯N and C—H⋯Cl inter­actions occur in the crystal structure.

## Related literature

For related literature, see: Kerbs (2003 ▶); Gude *et al.* (2005 ▶).
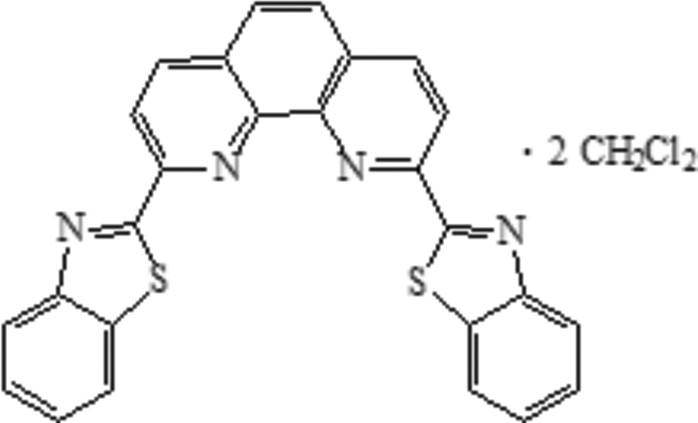


## Experimental

### 

#### Crystal data

C_26_H_14_N_4_S_2_·2CH_2_Cl_2_
*M* *_r_* = 616.38
Triclinic, 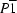

*a* = 8.0969 (2) Å
*b* = 12.3990 (2) Å
*c* = 14.6006 (3) Å
α = 108.234 (1)°
β = 102.181 (1)°
γ = 94.335 (1)°
*V* = 1344.93 (5) Å^3^
*Z* = 2
Cu *K*α radiation
μ = 5.67 mm^−1^
*T* = 100 (2) K
0.75 × 0.07 × 0.05 mm

#### Data collection

Bruker APEXII CCD diffractometer
Absorption correction: multi-scan (*SADABS*; Bruker, 2005 ▶) *T*_min_ = 0.101, *T*_max_ = 0.765
10764 measured reflections
4352 independent reflections
3831 reflections with *I* > 2σ(*I*)
*R*_int_ = 0.021

#### Refinement

*R*[*F* ^2^ > 2σ(*F* ^2^)] = 0.031
*wR*(*F* ^2^) = 0.079
*S* = 1.00
4352 reflections
415 parameters
All H-atom parameters refined
Δρ_max_ = 0.37 e Å^−3^
Δρ_min_ = −0.38 e Å^−3^

### 

Data collection: *APEX2* (Bruker, 2005 ▶); cell refinement: *SAINT* (Bruker, 2005 ▶); data reduction: *SAINT*; program(s) used to solve structure: *SHELXS97* (Sheldrick, 2008 ▶); program(s) used to refine structure: *SHELXL97* (Sheldrick, 2008 ▶); molecular graphics: *XP* (Bruker, 2005 ▶); software used to prepare material for publication: *XCIF* (Bruker, 2005 ▶).

## Supplementary Material

Crystal structure: contains datablocks I, global. DOI: 10.1107/S1600536808019995/hb2737sup1.cif

Structure factors: contains datablocks I. DOI: 10.1107/S1600536808019995/hb2737Isup2.hkl

Additional supplementary materials:  crystallographic information; 3D view; checkCIF report

## Figures and Tables

**Table 1:** Hydrogen-bond geometry (Å, °)

*D*—H⋯*A*	*D*—H	H⋯*A*	*D*⋯*A*	*D*—H⋯*A*
C1S—H1S*B*⋯N4^i^	0.94 (2)	2.44 (2)	3.360 (3)	166.7 (19)
C3—H3⋯Cl1S^ii^	0.92 (2)	2.82 (2)	3.615 (2)	145.6 (16)

Symmetry codes: (i) 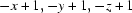; (ii) 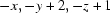.

**Fig. 1.**
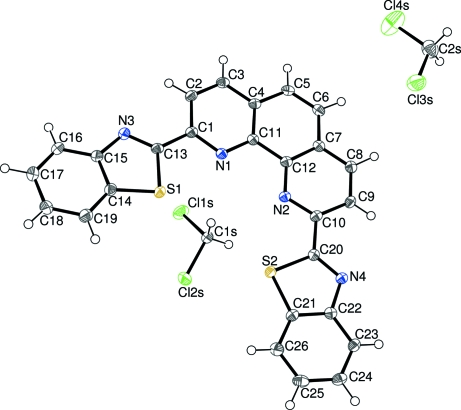
The molecular structure of (I) showing 50% displacement ellipsoids for the non-hydrogen atoms.

**Fig. 2.**
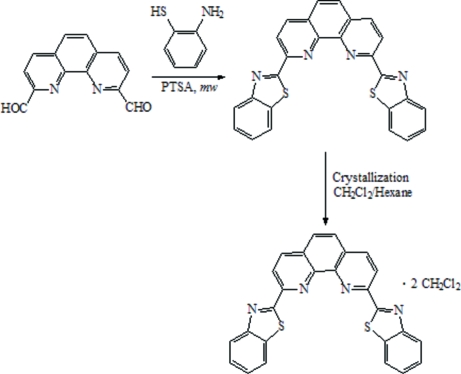
The formation of the title compound.

## supplementary crystallographic information

### Comment

As part of our studies of the biological properties of Schiff bases, we attempted to synthesize Schiff bases from 1,10-phenanthroline. It has been found in the literature that Schiff bases formed from S-alkyl and S-aryl substituted amines contain both hard nitrogen and soft sulfur donor atoms. (e.g. Kerbs, 2003). Consequently, these compounds are capable of forming stable complexes with a wide variety of metal ions.  These complexes have interesting physio-chemical properties and potential chemotherapeutic effets (e.g. Gude *et al.*2005). In this paper the synthesis and structure of the title compound, (I), are reported.

The main molecule is close to planar, with dihedral angles of 9.05 (5)° and 1.03 (7)° for the S1 and S2 benzothiazolyl moieties respectively, with respect to the phenanthroline core.  There are two dichloromethane solvent molecules in the asymmetric unit (Fig. 1).

Weak intermolecular C—H···N and C—H···Cl interactions (Table 1) may help to stabilise the packing.

### Experimental

To a solution of 1,10-phenanthroline (50 mg, 0.20 mmol) in 5 ml CHCl_3_, 2-mercaptoaniline (0.60 µ*L*, 0.40 mmol) was added followed by the addition of p-toluene sulfonic acid mono hydrate (76 mg, 0.40 mmol) in a Pyrex tube under argon. The tube was placed in a CEM microwave. The reaction conditions were set up as follows: power: 300 W, ramp time: 20 min, hold time: 20 min, and temperature: 373 K. When the reaction vessel was opened, a yellow precipitate was observed, which was filtered off and washed with cold CHCl_3_ and dried under vacuum. [*y*: 30 mg, 40%]. IR: ν= 1597 cm-1 (C=N), 1550 cm-1 (C=C). 1*H*-NMR([D_3_],CDCl_3_, 300 MHz): δ=8.74 (d, ^4^J= 9.0 Hz,, 8-H, 3-H), 8.41 (d, ^3^J=9.0 Hz,2*H*, H-4, H-7), 7.88 (s, 2H, H-5, and H-6), 8.169 (t, ^4^H, 2 J= 8.1 Hz, ^1^J=8.7 Hz,H-16, H-16', H-13, H-13'), 7.53 (m, 4H, H-14, H-14', H-15, H-15').13 C: NMR([D_3_], CDCl_3_, 75.5 MHz): δ= 155 (C-2, C-9), δ= 146 (C-11,*C*-12), δ=137.48 (C-3, C-8). δ=127.591 (C-4, C-7,), δ=126.60 (C-5, C-6), δ= 172 (C-13, C-13'), δ= 152 (C-14, C-14'), δ= 130.26 (C-15, C-15'), δ=137.0 (C-19, C-19') δ=126.18 (C-19, C-19'). δ=124.04 (C-18, C-18'). δ=122.54 (C-17, C-17'). δ=120.51 (C-16, C-16').

Yellow needles of (I) were grown from CH_2_Cl_2_/hexane at 253 K.

### Refinement

The H atoms were located in difference maps and their positions and U_iso_ values were freely refined.

### Crystal data

**Table:**

C_26_H_14_N_4_S_2_·2CH_2_Cl_2_	*Z* = 2
*M**_r_* = 616.38	*F*_000_ = 628
Triclinic, *P*1	*D*_x_ = 1.522 Mg m^−^^3^
Hall symbol: -P 1	Melting point > 573 K
*a* = 8.0969 (2) Å	Cu *K*α radiation λ = 1.54178 Å
*b* = 12.3990 (2) Å	Cell parameters from 5710 reflections
*c* = 14.6006 (3) Å	θ = 3–65º
α = 108.234 (1)º	µ = 5.67 mm^−^^1^
β = 102.181 (1)º	*T* = 100 (2) K
γ = 94.335 (1)º	Needle, yellow
*V* = 1344.93 (5) Å^3^	0.75 × 0.07 × 0.05 mm

### Data collection

**Table:**

Bruker APEXII CCD diffractometer	4352 independent reflections
Radiation source: fine-focus sealed tube	3831 reflections with *I* > 2σ(*I*)
Monochromator: graphite	*R*_int_ = 0.021
*T* = 100(2) K	θ_max_ = 66.5º
ω scans	θ_min_ = 3.3º
Absorption correction: multi-scan(SADABS; Bruker, 2005)	*h* = −9→9
*T*_min_ = 0.101, *T*_max_ = 0.765	*k* = −14→13
10764 measured reflections	*l* = 0→17

### Refinement

**Table:**

Refinement on *F*^2^	Secondary atom site location: difference Fourier map
Least-squares matrix: full	Hydrogen site location: difference Fourier map
*R*[*F*^2^ > 2σ(*F*^2^)] = 0.031	All H-atom parameters refined
*wR*(*F*^2^) = 0.079	*w* = 1/[σ^2^(*F*_o_^2^) + (0.0482*P*)^2^ + 0.6095*P*] where *P* = (*F*_o_^2^ + 2*F*_c_^2^)/3
*S* = 1.00	(Δ/σ)_max_ = 0.001
4352 reflections	Δρ_max_ = 0.37 e Å^−^^3^
415 parameters	Δρ_min_ = −0.38 e Å^−^^3^
Primary atom site location: structure-invariant direct methods	Extinction correction: none

### Special details

**Table:**

Geometry. All e.s.d.'s (except the e.s.d. in the dihedral angle between two l.s. planes) are estimated using the full covariance matrix. The cell e.s.d.'s are taken into account individually in the estimation of e.s.d.'s in distances, angles and torsion angles; correlations between e.s.d.'s in cell parameters are only used when they are defined by crystal symmetry. An approximate (isotropic) treatment of cell e.s.d.'s is used for estimating e.s.d.'s involving l.s. planes.
Refinement. Refinement of *F*^2^ against ALL reflections. The weighted *R*-factor *wR* and goodness of fit *S* are based on *F*^2^, conventional *R*-factors *R* are based on *F*, with *F* set to zero for negative *F*^2^. The threshold expression of *F*^2^ > σ(*F*^2^) is used only for calculating *R*-factors(gt) *etc*. and is not relevant to the choice of reflections for refinement. *R*-factors based on *F*^2^ are statistically about twice as large as those based on *F*, and *R*- factors based on ALL data will be even larger.

### Fractional atomic coordinates and isotropic or equivalent isotropic displacement parameters (Å^2^)

**Table:**

	*x*	*y*	*z*	*U*_iso_*/*U*_eq_	
S1	−0.16197 (6)	0.64055 (4)	0.22928 (3)	0.02003 (12)	
S2	0.22351 (6)	0.40021 (4)	0.33879 (3)	0.01732 (12)	
N1	−0.02010 (19)	0.73385 (13)	0.44545 (11)	0.0161 (3)	
N2	0.17914 (18)	0.58715 (12)	0.50867 (11)	0.0155 (3)	
N3	−0.2641 (2)	0.84086 (13)	0.26682 (12)	0.0211 (4)	
N4	0.39069 (19)	0.33830 (12)	0.48236 (11)	0.0167 (3)	
C1	−0.1189 (2)	0.80420 (15)	0.41677 (14)	0.0172 (4)	
C2	−0.1611 (2)	0.90203 (16)	0.48337 (14)	0.0195 (4)	
C3	−0.0951 (2)	0.92663 (16)	0.58255 (14)	0.0195 (4)	
C4	0.0093 (2)	0.85441 (15)	0.61687 (13)	0.0164 (4)	
C5	0.0753 (2)	0.87447 (16)	0.72043 (14)	0.0186 (4)	
C6	0.1658 (2)	0.79970 (16)	0.75169 (14)	0.0196 (4)	
C7	0.2011 (2)	0.69974 (15)	0.68113 (13)	0.0167 (4)	
C8	0.2901 (2)	0.61883 (16)	0.71135 (14)	0.0188 (4)	
C9	0.3234 (2)	0.52576 (16)	0.64199 (14)	0.0180 (4)	
C10	0.2672 (2)	0.51431 (15)	0.54114 (14)	0.0160 (4)	
C11	0.0425 (2)	0.75722 (15)	0.54414 (13)	0.0156 (4)	
C12	0.1452 (2)	0.67863 (15)	0.57772 (13)	0.0159 (4)	
C13	−0.1855 (2)	0.77490 (15)	0.30927 (14)	0.0174 (4)	
C14	−0.2697 (2)	0.67676 (16)	0.12950 (14)	0.0198 (4)	
C15	−0.3127 (2)	0.78754 (16)	0.16477 (14)	0.0202 (4)	
C16	−0.3991 (3)	0.83493 (19)	0.09613 (15)	0.0274 (5)	
C17	−0.4411 (3)	0.77107 (19)	−0.00363 (16)	0.0295 (5)	
C18	−0.3985 (3)	0.66055 (19)	−0.03754 (16)	0.0280 (5)	
C19	−0.3121 (3)	0.61241 (18)	0.02860 (15)	0.0247 (4)	
C20	0.3034 (2)	0.41652 (15)	0.46417 (13)	0.0154 (4)	
C21	0.3131 (2)	0.27520 (15)	0.30739 (14)	0.0177 (4)	
C22	0.3991 (2)	0.25688 (15)	0.39417 (14)	0.0171 (4)	
C23	0.4800 (2)	0.15927 (16)	0.38635 (15)	0.0196 (4)	
C24	0.4739 (2)	0.08355 (16)	0.29330 (15)	0.0221 (4)	
C25	0.3865 (3)	0.10262 (17)	0.20755 (15)	0.0245 (4)	
C26	0.3058 (2)	0.19776 (17)	0.21311 (15)	0.0219 (4)	
H2	−0.233 (3)	0.9445 (19)	0.4549 (16)	0.028 (6)*	
H3	−0.118 (2)	0.9906 (18)	0.6279 (15)	0.017 (5)*	
H5	0.054 (2)	0.9395 (18)	0.7645 (15)	0.016 (5)*	
H6	0.207 (2)	0.8083 (16)	0.8204 (15)	0.015 (5)*	
H8	0.328 (2)	0.6297 (17)	0.7835 (16)	0.021 (5)*	
H9	0.380 (3)	0.4734 (18)	0.6609 (15)	0.022 (5)*	
H16	−0.425 (3)	0.909 (2)	0.1181 (16)	0.029 (6)*	
H17	−0.504 (3)	0.803 (2)	−0.0520 (17)	0.034 (6)*	
H18	−0.429 (2)	0.6211 (17)	−0.1043 (16)	0.019 (5)*	
H19	−0.286 (3)	0.535 (2)	0.0041 (16)	0.029 (6)*	
H23	0.535 (3)	0.1458 (18)	0.4413 (16)	0.021 (5)*	
H24	0.528 (3)	0.0190 (19)	0.2872 (15)	0.023 (5)*	
H25	0.381 (3)	0.0496 (19)	0.1426 (16)	0.025 (6)*	
H26	0.245 (3)	0.2090 (17)	0.1567 (16)	0.021 (5)*	
Cl1S	0.32173 (7)	0.83312 (4)	0.34294 (4)	0.03355 (14)	
Cl2S	0.32540 (7)	0.60371 (4)	0.20953 (4)	0.03418 (14)	
C1S	0.3093 (3)	0.68551 (17)	0.33012 (15)	0.0217 (4)	
H1SA	0.201 (3)	0.6616 (17)	0.3398 (14)	0.020 (5)*	
H1SB	0.403 (3)	0.6741 (18)	0.3742 (16)	0.026 (6)*	
Cl3S	0.07322 (8)	0.63525 (5)	0.93119 (5)	0.04547 (17)	
Cl4S	0.05564 (10)	0.87470 (6)	1.03272 (6)	0.0674 (2)	
C2S	0.1165 (3)	0.7470 (2)	1.04721 (19)	0.0406 (6)	
H2SA	0.241 (4)	0.763 (2)	1.0784 (19)	0.049 (7)*	
H2SB	0.047 (3)	0.726 (2)	1.087 (2)	0.049 (8)*	

### Atomic displacement parameters (Å^2^)

**Table:**

	*U*^11^	*U*^22^	*U*^33^	*U*^12^	*U*^13^	*U*^23^
S1	0.0242 (3)	0.0178 (2)	0.0193 (2)	0.00770 (18)	0.00485 (18)	0.00731 (19)
S2	0.0174 (2)	0.0169 (2)	0.0178 (2)	0.00438 (17)	0.00378 (17)	0.00611 (18)
N1	0.0145 (8)	0.0172 (8)	0.0189 (8)	0.0021 (6)	0.0067 (6)	0.0076 (6)
N2	0.0128 (8)	0.0152 (8)	0.0189 (8)	0.0011 (6)	0.0053 (6)	0.0058 (6)
N3	0.0232 (9)	0.0223 (9)	0.0222 (9)	0.0082 (7)	0.0080 (7)	0.0109 (7)
N4	0.0146 (8)	0.0159 (8)	0.0204 (8)	0.0020 (6)	0.0054 (6)	0.0066 (6)
C1	0.0142 (9)	0.0175 (9)	0.0226 (10)	0.0019 (7)	0.0076 (7)	0.0085 (8)
C2	0.0198 (10)	0.0174 (9)	0.0251 (10)	0.0062 (8)	0.0087 (8)	0.0097 (8)
C3	0.0205 (10)	0.0154 (10)	0.0241 (10)	0.0028 (8)	0.0100 (8)	0.0057 (8)
C4	0.0136 (9)	0.0153 (9)	0.0210 (10)	0.0000 (7)	0.0073 (7)	0.0055 (7)
C5	0.0174 (10)	0.0163 (10)	0.0206 (10)	0.0009 (7)	0.0079 (7)	0.0025 (8)
C6	0.0180 (10)	0.0232 (10)	0.0168 (10)	0.0010 (8)	0.0053 (7)	0.0054 (8)
C7	0.0124 (9)	0.0185 (9)	0.0185 (9)	−0.0010 (7)	0.0041 (7)	0.0059 (8)
C8	0.0144 (9)	0.0225 (10)	0.0203 (10)	0.0009 (7)	0.0045 (7)	0.0086 (8)
C9	0.0149 (10)	0.0186 (10)	0.0224 (10)	0.0038 (8)	0.0040 (7)	0.0098 (8)
C10	0.0104 (9)	0.0148 (9)	0.0233 (10)	0.0002 (7)	0.0054 (7)	0.0069 (8)
C11	0.0121 (9)	0.0162 (9)	0.0201 (10)	−0.0005 (7)	0.0060 (7)	0.0076 (7)
C12	0.0119 (9)	0.0155 (9)	0.0209 (10)	−0.0006 (7)	0.0063 (7)	0.0062 (8)
C13	0.0161 (10)	0.0169 (9)	0.0222 (10)	0.0032 (7)	0.0093 (7)	0.0078 (8)
C14	0.0163 (10)	0.0237 (10)	0.0228 (10)	0.0051 (8)	0.0065 (7)	0.0110 (8)
C15	0.0203 (10)	0.0228 (10)	0.0206 (10)	0.0065 (8)	0.0077 (7)	0.0089 (8)
C16	0.0330 (12)	0.0290 (12)	0.0264 (11)	0.0177 (9)	0.0105 (9)	0.0128 (9)
C17	0.0310 (12)	0.0381 (13)	0.0238 (11)	0.0151 (10)	0.0061 (9)	0.0145 (10)
C18	0.0301 (12)	0.0337 (12)	0.0187 (11)	0.0105 (9)	0.0040 (8)	0.0069 (9)
C19	0.0260 (11)	0.0233 (11)	0.0239 (11)	0.0069 (8)	0.0048 (8)	0.0068 (9)
C20	0.0109 (9)	0.0163 (9)	0.0199 (9)	−0.0003 (7)	0.0042 (7)	0.0077 (7)
C21	0.0136 (9)	0.0165 (9)	0.0235 (10)	0.0017 (7)	0.0053 (7)	0.0072 (8)
C22	0.0141 (9)	0.0163 (9)	0.0209 (10)	−0.0011 (7)	0.0057 (7)	0.0062 (8)
C23	0.0174 (10)	0.0185 (10)	0.0271 (11)	0.0030 (7)	0.0080 (8)	0.0116 (8)
C24	0.0206 (10)	0.0155 (10)	0.0323 (12)	0.0028 (8)	0.0115 (8)	0.0077 (8)
C25	0.0237 (11)	0.0199 (10)	0.0254 (11)	0.0005 (8)	0.0086 (8)	0.0005 (9)
C26	0.0182 (10)	0.0241 (10)	0.0201 (10)	0.0017 (8)	0.0027 (8)	0.0045 (8)
Cl1S	0.0412 (3)	0.0209 (3)	0.0436 (3)	0.0111 (2)	0.0151 (2)	0.0133 (2)
Cl2S	0.0537 (4)	0.0306 (3)	0.0227 (3)	0.0168 (2)	0.0146 (2)	0.0094 (2)
C1S	0.0249 (11)	0.0205 (10)	0.0228 (11)	0.0081 (8)	0.0076 (8)	0.0095 (8)
Cl3S	0.0506 (4)	0.0385 (3)	0.0509 (4)	0.0138 (3)	0.0195 (3)	0.0139 (3)
Cl4S	0.0650 (5)	0.0360 (4)	0.0686 (5)	0.0069 (3)	−0.0205 (4)	−0.0029 (3)
C2S	0.0348 (14)	0.0513 (15)	0.0376 (14)	0.0033 (11)	0.0082 (11)	0.0187 (12)

### Geometric parameters (Å, °)

**Table:**

S1—C14	1.7370 (19)	C10—C20	1.469 (2)
S1—C13	1.7607 (18)	C11—C12	1.460 (2)
S2—C21	1.7334 (18)	C14—C19	1.391 (3)
S2—C20	1.7491 (18)	C14—C15	1.407 (3)
N1—C1	1.330 (2)	C15—C16	1.405 (3)
N1—C11	1.351 (2)	C16—C17	1.376 (3)
N2—C10	1.331 (2)	C16—H16	0.94 (2)
N2—C12	1.352 (2)	C17—C18	1.400 (3)
N3—C13	1.299 (2)	C17—H17	0.98 (2)
N3—C15	1.382 (2)	C18—C19	1.385 (3)
N4—C20	1.303 (2)	C18—H18	0.91 (2)
N4—C22	1.384 (2)	C19—H19	0.97 (2)
C1—C2	1.414 (3)	C21—C26	1.397 (3)
C1—C13	1.464 (3)	C21—C22	1.405 (3)
C2—C3	1.359 (3)	C22—C23	1.404 (3)
C2—H2	0.93 (2)	C23—C24	1.377 (3)
C3—C4	1.408 (3)	C23—H23	0.90 (2)
C3—H3	0.92 (2)	C24—C25	1.400 (3)
C4—C11	1.422 (3)	C24—H24	0.93 (2)
C4—C5	1.427 (3)	C25—C26	1.380 (3)
C5—C6	1.353 (3)	C25—H25	0.96 (2)
C5—H5	0.92 (2)	C26—H26	0.92 (2)
C6—C7	1.435 (3)	Cl1S—C1S	1.7733 (19)
C6—H6	0.96 (2)	Cl2S—C1S	1.773 (2)
C7—C8	1.405 (3)	C1S—H1SA	0.95 (2)
C7—C12	1.414 (3)	C1S—H1SB	0.94 (2)
C8—C9	1.364 (3)	Cl3S—C2S	1.766 (3)
C8—H8	0.99 (2)	Cl4S—C2S	1.754 (3)
C9—C10	1.404 (3)	C2S—H2SA	0.99 (3)
C9—H9	0.90 (2)	C2S—H2SB	0.97 (3)
			
C14—S1—C13	88.49 (9)	N3—C15—C16	125.46 (18)
C21—S2—C20	88.48 (9)	N3—C15—C14	115.44 (17)
C1—N1—C11	117.85 (15)	C16—C15—C14	119.10 (18)
C10—N2—C12	117.47 (15)	C17—C16—C15	119.06 (19)
C13—N3—C15	110.46 (16)	C17—C16—H16	120.7 (14)
C20—N4—C22	110.30 (15)	C15—C16—H16	120.3 (14)
N1—C1—C2	123.86 (17)	C16—C17—C18	121.17 (19)
N1—C1—C13	116.02 (16)	C16—C17—H17	119.5 (13)
C2—C1—C13	120.12 (16)	C18—C17—H17	119.3 (13)
C3—C2—C1	118.15 (18)	C19—C18—C17	120.83 (19)
C3—C2—H2	125.4 (14)	C19—C18—H18	120.8 (13)
C1—C2—H2	116.4 (14)	C17—C18—H18	118.4 (13)
C2—C3—C4	120.23 (18)	C18—C19—C14	118.09 (19)
C2—C3—H3	120.3 (12)	C18—C19—H19	119.8 (13)
C4—C3—H3	119.5 (12)	C14—C19—H19	122.1 (13)
C3—C4—C11	117.41 (17)	N4—C20—C10	124.42 (16)
C3—C4—C5	122.00 (17)	N4—C20—S2	116.40 (14)
C11—C4—C5	120.56 (17)	C10—C20—S2	119.17 (13)
C6—C5—C4	120.92 (17)	C26—C21—C22	121.22 (17)
C6—C5—H5	121.6 (12)	C26—C21—S2	128.96 (15)
C4—C5—H5	117.4 (12)	C22—C21—S2	109.82 (14)
C5—C6—C7	120.55 (17)	N4—C22—C23	125.39 (17)
C5—C6—H6	123.3 (12)	N4—C22—C21	114.98 (16)
C7—C6—H6	116.1 (12)	C23—C22—C21	119.60 (17)
C8—C7—C12	117.46 (16)	C24—C23—C22	119.06 (19)
C8—C7—C6	121.80 (17)	C24—C23—H23	120.1 (13)
C12—C7—C6	120.73 (16)	C22—C23—H23	120.9 (13)
C9—C8—C7	120.03 (17)	C23—C24—C25	120.61 (18)
C9—C8—H8	120.5 (12)	C23—C24—H24	119.8 (13)
C7—C8—H8	119.5 (12)	C25—C24—H24	119.6 (13)
C8—C9—C10	118.20 (17)	C26—C25—C24	121.51 (19)
C8—C9—H9	120.5 (13)	C26—C25—H25	117.7 (13)
C10—C9—H9	121.3 (13)	C24—C25—H25	120.8 (13)
N2—C10—C9	124.03 (17)	C25—C26—C21	117.99 (19)
N2—C10—C20	116.04 (16)	C25—C26—H26	121.5 (13)
C9—C10—C20	119.93 (16)	C21—C26—H26	120.4 (13)
N1—C11—C4	122.47 (16)	Cl2S—C1S—Cl1S	110.15 (11)
N1—C11—C12	118.95 (16)	Cl2S—C1S—H1SA	109.8 (12)
C4—C11—C12	118.57 (16)	Cl1S—C1S—H1SA	107.6 (12)
N2—C12—C7	122.76 (16)	Cl2S—C1S—H1SB	105.3 (13)
N2—C12—C11	118.65 (16)	Cl1S—C1S—H1SB	110.1 (13)
C7—C12—C11	118.56 (16)	H1SA—C1S—H1SB	113.8 (18)
N3—C13—C1	124.79 (16)	Cl4S—C2S—Cl3S	111.12 (14)
N3—C13—S1	116.14 (14)	Cl4S—C2S—H2SA	106.9 (15)
C1—C13—S1	119.06 (13)	Cl3S—C2S—H2SA	109.9 (15)
C19—C14—C15	121.75 (18)	Cl4S—C2S—H2SB	106.1 (16)
C19—C14—S1	128.78 (15)	Cl3S—C2S—H2SB	108.2 (16)
C15—C14—S1	109.46 (14)	H2SA—C2S—H2SB	115 (2)
			
C11—N1—C1—C2	0.4 (3)	C14—S1—C13—N3	−0.46 (15)
C11—N1—C1—C13	−179.31 (15)	C14—S1—C13—C1	178.90 (14)
N1—C1—C2—C3	0.9 (3)	C13—S1—C14—C19	−179.45 (19)
C13—C1—C2—C3	−179.40 (16)	C13—S1—C14—C15	0.63 (14)
C1—C2—C3—C4	−1.3 (3)	C13—N3—C15—C16	−179.48 (19)
C2—C3—C4—C11	0.5 (3)	C13—N3—C15—C14	0.4 (2)
C2—C3—C4—C5	−177.33 (17)	C19—C14—C15—N3	179.34 (17)
C3—C4—C5—C6	175.55 (18)	S1—C14—C15—N3	−0.7 (2)
C11—C4—C5—C6	−2.2 (3)	C19—C14—C15—C16	−0.8 (3)
C4—C5—C6—C7	1.5 (3)	S1—C14—C15—C16	179.16 (15)
C5—C6—C7—C8	−177.99 (17)	N3—C15—C16—C17	−179.27 (19)
C5—C6—C7—C12	1.3 (3)	C14—C15—C16—C17	0.8 (3)
C12—C7—C8—C9	1.7 (3)	C15—C16—C17—C18	−0.4 (3)
C6—C7—C8—C9	−179.01 (17)	C16—C17—C18—C19	−0.2 (3)
C7—C8—C9—C10	0.3 (3)	C17—C18—C19—C14	0.3 (3)
C12—N2—C10—C9	1.2 (2)	C15—C14—C19—C18	0.2 (3)
C12—N2—C10—C20	−179.38 (15)	S1—C14—C19—C18	−179.70 (16)
C8—C9—C10—N2	−1.8 (3)	C22—N4—C20—C10	178.79 (16)
C8—C9—C10—C20	178.81 (16)	C22—N4—C20—S2	−0.35 (19)
C1—N1—C11—C4	−1.3 (2)	N2—C10—C20—N4	178.51 (16)
C1—N1—C11—C12	177.38 (16)	C9—C10—C20—N4	−2.1 (3)
C3—C4—C11—N1	0.9 (3)	N2—C10—C20—S2	−2.4 (2)
C5—C4—C11—N1	178.72 (16)	C9—C10—C20—S2	177.03 (13)
C3—C4—C11—C12	−177.81 (16)	C21—S2—C20—N4	0.89 (14)
C5—C4—C11—C12	0.0 (2)	C21—S2—C20—C10	−178.30 (14)
C10—N2—C12—C7	0.9 (2)	C20—S2—C21—C26	177.75 (18)
C10—N2—C12—C11	−177.37 (15)	C20—S2—C21—C22	−1.12 (13)
C8—C7—C12—N2	−2.3 (3)	C20—N4—C22—C23	−178.48 (17)
C6—C7—C12—N2	178.33 (16)	C20—N4—C22—C21	−0.6 (2)
C8—C7—C12—C11	175.94 (16)	C26—C21—C22—N4	−177.75 (16)
C6—C7—C12—C11	−3.4 (2)	S2—C21—C22—N4	1.22 (19)
N1—C11—C12—N2	2.3 (2)	C26—C21—C22—C23	0.3 (3)
C4—C11—C12—N2	−178.98 (15)	S2—C21—C22—C23	179.26 (14)
N1—C11—C12—C7	−176.04 (15)	N4—C22—C23—C24	178.07 (17)
C4—C11—C12—C7	2.7 (2)	C21—C22—C23—C24	0.3 (3)
C15—N3—C13—C1	−179.20 (16)	C22—C23—C24—C25	−0.8 (3)
C15—N3—C13—S1	0.1 (2)	C23—C24—C25—C26	0.8 (3)
N1—C1—C13—N3	−170.20 (17)	C24—C25—C26—C21	−0.2 (3)
C2—C1—C13—N3	10.1 (3)	C22—C21—C26—C25	−0.3 (3)
N1—C1—C13—S1	10.5 (2)	S2—C21—C26—C25	−179.07 (15)
C2—C1—C13—S1	−169.22 (14)		

### Hydrogen-bond geometry (Å, °)

**Table:**

*D*—H···*A*	*D*—H	H···*A*	*D*···*A*	*D*—H···*A*
C1S—H1SB···N4^i^	0.94 (2)	2.44 (2)	3.360 (3)	166.7 (19)
C3—H3···Cl1S^ii^	0.92 (2)	2.82 (2)	3.615 (2)	145.6 (16)

Symmetry codes: (i) −*x*+1, −*y*+1, −*z*+1; (ii) −*x*, −*y*+2, −*z*+1.
